# Endolysins and membrane-active peptides: innovative engineering strategies against gram-negative bacteria

**DOI:** 10.3389/fmicb.2025.1603380

**Published:** 2025-06-03

**Authors:** Monika Wojciechowska

**Affiliations:** University of Warsaw, Centre of New Technologies, Warsaw, Poland

**Keywords:** endolysins, peptides, antimicrobial peptides, bacteriophage, antibiotics, gram-negative bacteria, antimicrobial agents

## Abstract

Endolysins, bacteriophage-encoded peptidoglycan hydrolases, offer promising potential in antibacterial therapy, including treatments targeting gram-negative bacteria. While these enzymes naturally act primarily on gram-positive bacteria, their application against gram-negative pathogens is more challenging due to the presence of a dual-layer cell membrane, which acts as a protective barrier. However, innovative approaches, such as fusing endolysins with antimicrobial peptides (AMPs), have demonstrated increased efficacy against gram-negative bacteria. Modifying endolysins by introducing hydrophobic properties or positive charges or combining them with agents that disrupt the outer membrane enhances their bactericidal activity. Moreover, phage endolysins that exhibit activity against gram-negative bacteria are a promising source of membrane-active peptides. Identifying new peptide sequences derived from endolysins capable of penetrating the bacterial cell membrane represents a novel and increasingly explored research direction. Studying these innovative strategies had yielded promising results, though the field remains under active investigation and development. Ongoing efforts aim to optimize these approaches to improve their effectiveness against antibiotic-resistant gram-negative bacterial strains, which are particularly difficult to treat with conventional antibiotics. This review summarizes the latest advancements and solutions in the field, highlighting the potential of endolysins and membrane-active peptides as next-generation antibacterial agents.

## 1 Introduction

**Figure 1:**
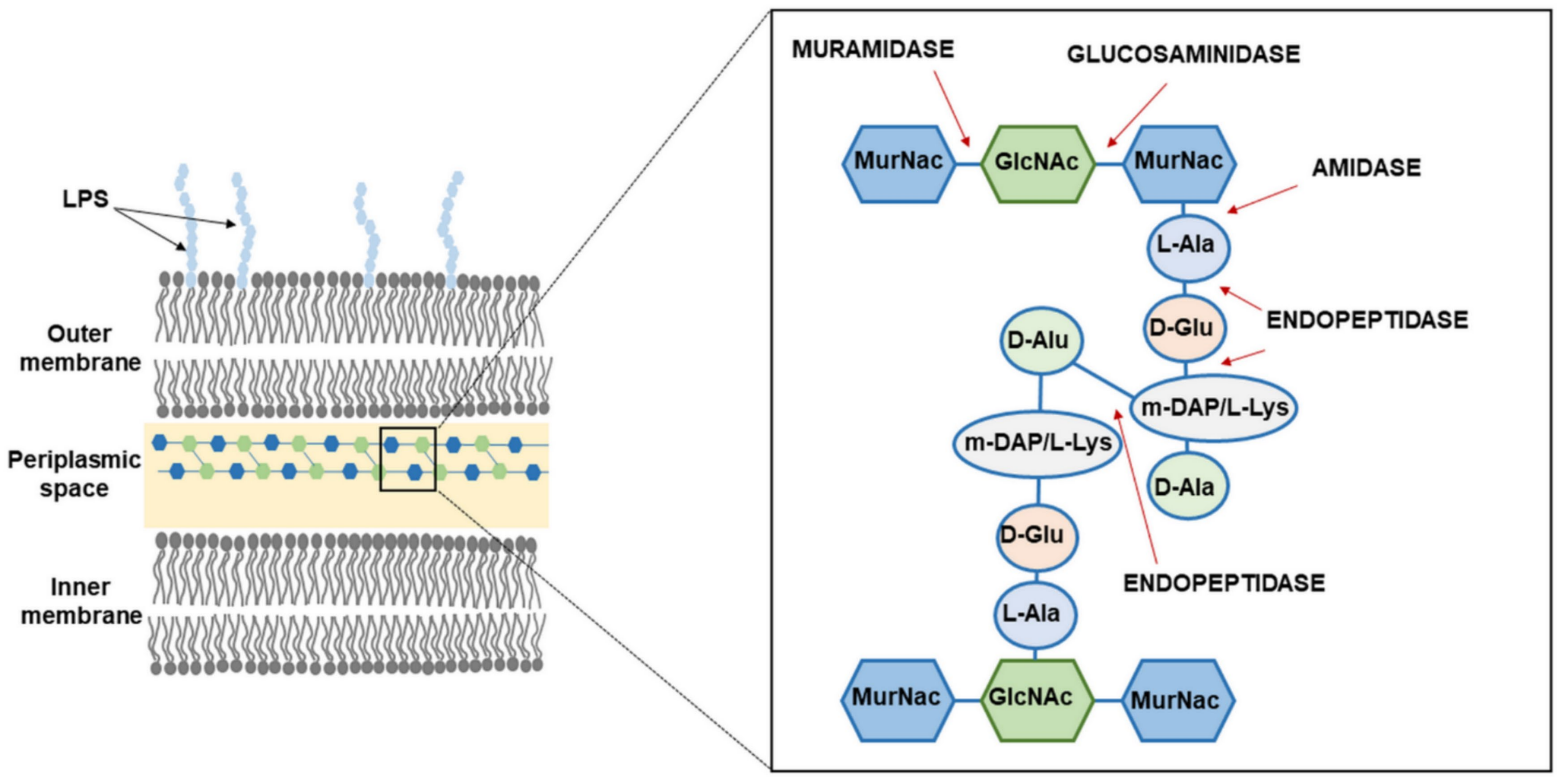
Schematic representation of the gram-negative cell wall and cleavage sites of bacteriophage endolysins in the peptidoglycan layer: MurNac, N-acetylmuramic acid; GlcNac, N-acetylglucosamine; m-DAP, diamino acid; L-Ala, L-alanine; D-Glu, D-glutamic acid; L-Lys; L-lysine; D-Ala, D-alanine; LPS, lipopolysaccharides.

The emergence of multidrug-resistant (MDR) bacteria has become a global health threat (Salam et al., 2023). The World Health Organization (WHO) has prioritized the fight against drug resistance (World Health Organization, 2017, 2024). Antibiotics are becoming increasingly ineffective as resistance spreads worldwide, leading to infections that are becoming difficult to treat. There is an urgent need to develop more potent, non-toxic, and effective antimicrobials against MDR strains (Parmanik et al., 2022). The WHO published a list of antibiotic-resistant “priority pathogens,” which include carbapenem-resistant *Acinetobacter baumannii* and *Pseudomonas aeruginosa*, as well as extended-spectrum β-lactamases (ESBL)—producing *Enterobacteriaceae* (World Health Organization, 2017, 2024). These gram-negative bacteria, responsible for severe and often deadly infections, such as systemic infections and pneumonia. These bacteria resist many antibiotics, including carbapenems and third-generation cephalosporins, which are considered the drugs of last resort in treating gram-negative bacteria-related infections. Additionally, gram-negative bacteria are the most frequent cause of food spoilage and contamination in industrial systems (Wdowiak et al., 2023). A major challenge in developing effective antibiotics against these pathogens is the low permeability of the gram-negative bacteria cell envelopes (Cama et al., 2019). Their outer membrane is an exceptionally sophisticated macromolecular barrier, providing an extra layer of protection while regulating material exchange (Figure 1). Beneath this lies a thin periplasmic space with peptidoglycan (PG) layer and a phospholipid-based inner membrane, essential for transport, biosynthesis, and DNA anchoring (Breijyeh et al., 2020; Alegun et al., 2021). Because of its crucial role in bacterial survival, the bacterial envelope has become a primary target for researchers seeking new antibiotics. One promising alternative to conventional approaches is to disrupt the gram-negative bacterial cell membrane, distorting its integrity and causing bacterial lysis.

Bacteriophages (phages) are the most abundant viruses on Earth and are natural antibacterial agents. Shortly after their discovery in 1919, phages were first used for therapeutic purposes in humans. One of their key advantages is their ability to evolve, allowing them to effectively combat multidrug-resistant pathogens and biofilms, combined with their specificity in targeting bacteria, which minimizes disruption to the host’s microbiota. However, a significant challenge to their therapeutic use is their rapid elimination by the human immune system, which limits their efficacy as antibiotics (Ghose and Euler, 2020; Rahman et al., 2021). One of the most promising approaches to overcome this obstacle is using phage-derived lytic enzymes, such as endolysins. These enzymes, responsible for bacterial lysis, are considered promising candidates for future antimicrobial therapies, particularly in combating antibiotic-resistant bacteria. Understanding their mechanisms of action could pave the way for their incorporation into commonly used disinfectants or therapeutic agents.

This review provides an integrated and up-to-date perspective on the engineering of endolysins for activity against gram-negative bacteria, with particular emphasis on membrane-targeting strategies. While several recent reviews have addressed individual aspects of endolysin activity, AMP fusion, or synergy with antibiotics, this manuscript offers a comprehensive synthesis of modular engineering approaches, including the use of synthetic linkers, lipid tails, nanocarriers, and Trojan-horse delivery designs. Additionally, it summarizes the development of peptides with outer membrane-disrupting activity derived from endolysin sequences. Recent advances in combinatorial design platforms such as VersaTile and the application of artificial intelligence tools to identify hidden antimicrobial motifs within lysin sequences are also highlighted. By integrating insights from endolysin biology and AMP engineering, this review outlines the current challenges and future directions in the development of endolysin-based therapeutics against multidrug-resistant gram-negative pathogens.

## 2 Endolysins—structure and activity

The term “endolysins” was first introduced in 1958 to describe bacteriophage-encoded peptidoglycan hydrolases (Jacob and Fuerst, 1958). These enzymes are synthesized within bacterial cells infected by a phage during the late stages of the phage replication cycle. Their primary function is to degrade the bacterial PG layer, facilitating host cell lysis and the subsequent release of newly assembled phage particles. Larger bacteriophages with a double-stranded DNA genome encode at least two lytic proteins necessary for bacterial cell lysis: holin and endolysin. Holin accumulates in the bacterial cytoplasmic membrane and, at a precisely programmed time, forms lethal pores that increase membrane permeability. This process allows endolysin to move from the cytoplasm into the bacterial periplasm, where it degrades the PG layer (Saier and Reddy, 2015; Cahill and Young, 2019; Bai et al., 2020; Xu et al., 2021; Pattnaik et al., 2024).

**Figure 2:**
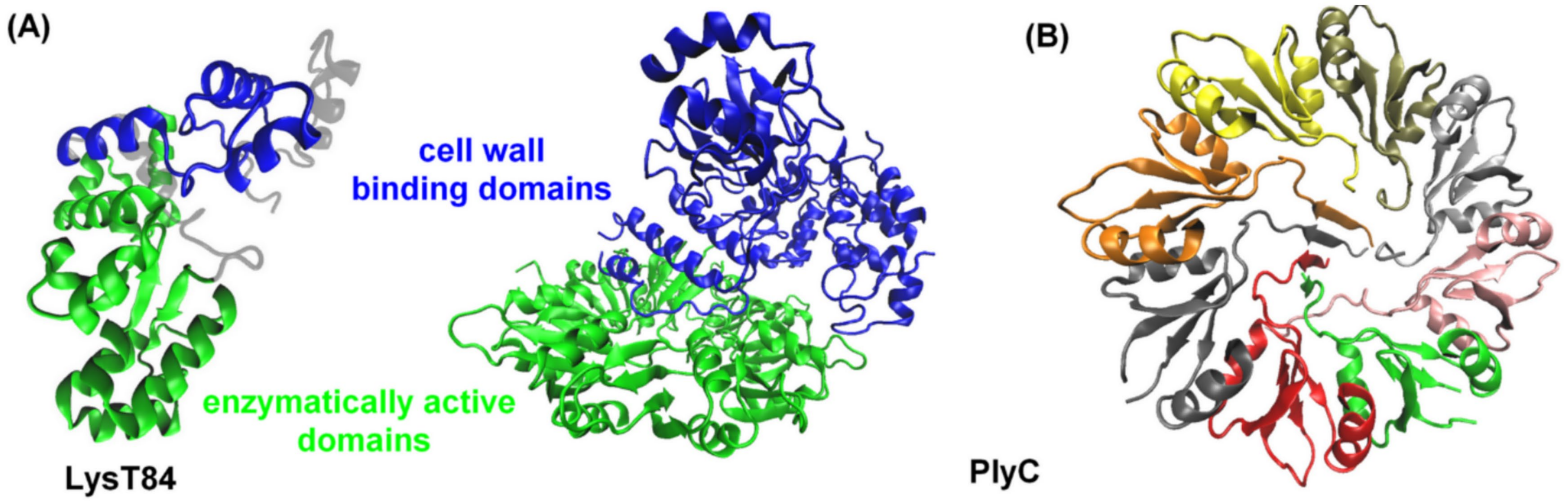
**(A)** Three-dimensional structures of the LysT84 (PDB code: 7RUM, Love et al., 2022) and PlyC (PDB code: 4F88, McGowan et al., 2012) endolysins. Enzymatically active domains are shown in green, while cell wall binding domains are depicted in blue. **(B)** Representation of the cell wall binding surface of PlyC endolysin, divided into eight domains. The proteins are displayed in a cartoon representation, using VMD (Humphrey et al., 1996).

The endolysins of phages that infect gram-negative bacteria are typically simple, globular proteins of 15–20 kDa, consisting predominantly of an enzymatically active domain (EAD) (Rahman et al., 2021). However, some endolysins possess more than one EAD. Exceptions include two endolysins from *P. aeruginosa* phages KZ144 and EL188, which display a modular structure featuring an N-terminal cell wall binding domain (CBD) and a C-terminal EAD (Figure 2; Briers et al., 2007, 2009, 2011). Other endolysins with a modular structure (one CBD and one EAD), that target gram-negative bacteria, include *Salmonella enterica* bacteriophage endolysin Gp110 (Rodríguez-Rubio et al., 2016) and *E. coli* bacteriophage endolysin LysT84 (Figure 2; Love et al., 2022). The structure of endolysins may vary depending on the type of bacteria they target, e.g., the PlyC enzyme of bacteriophages targeting *Streptococcus* spp. is a holoenzyme consisting of nine subunits: one catalytic subunit with both glycosidase and endopeptidase activity and eight identical CBD subunits (Figure 2B; McGowan et al., 2012). This modular architecture was initially thought to be unique to endolysins from phages targeting gram-positive bacteria, where it is often arranged in the reverse orientation. Unlike gram-negative bacteria, gram-positive bacteria lack a protective outer membrane and have a thick, multi-layered PG cell wall adorned with surface carbohydrates, proteins, and wall teichoic acids (Rohde, 2019). This structural difference may explain the modular architecture of endolysins from gram-positive bacterial phages. In contrast, the presence of an outer membrane in gram-negative bacteria reduces the necessity for an additional CBD. Additionally, the thinner PG layer in gram-negative bacteria may not require as much enzymatic anchoring as the multilayered PG of gram-positive bacterial cell walls.

Bacterial PG is composed of two major amino-sugar components: N-acetylglucosamine and N-acetylmuramic acid, connected by a β-(1,4)-glycosidic bond. This polysaccharide lattice is stabilized by amino acid chains, whose composition differs between gram-positive and gram-negative bacteria. Their common feature is the presence of L-alanine and D-alanine (Garde et al., 2021). Since this structure is highly conserved in both of these bacterial types, the number of covalent bonds that endolysins or other PG-digesting enzymes can target is inherently limited. The EAD of these enzymes specializes in cleaving one of the four primary bonds within the PG structure. Therefore, based on their activity, endolysins were classified into four main groups: glucosaminidase, amidases, endopeptidases, and muramidase (Figure 1; Gerstmans et al., 2018; Vázquez et al., 2018; Liu B. et al., 2023; Khan et al., 2024; Pattnaik et al., 2024; Zheng and Zhang, 2024). Glycosidases cleave bonds between N-acetylmuramic acid (MurNAc) and N-acetylglucosamine (GlcNAc) in the carbohydrate chains of PG. They can be divided into two types: N-acetylglucosaminidases, which hydrolyze the β-1,4-glycosidic bond at the GlcNAc end, and N-acetylmuramidases, which target glycosidic bonds at the MurNAc end. The second group, amidohydrolases (amidases), cleaves the amide bond between MurNAc and the peptide residue (L-alanine) in bacterial PG. The third group, endopeptidases (or endoproteases), hydrolyzes peptide bonds within core peptides attached to MurNAc or those forming cross-links, disrupting PG structural integrity. The fourth group, lytic transglycosylases, like muramidases, digest β-1,4-glycosidic bonds between MurNAc and GlcNAc residues. Among the studied endolysins, most had been classified as amidases or muramidases (Gerstmans et al., 2018; Vázquez et al., 2018; Liu B. et al., 2023; Khan et al., 2024; Pattnaik et al., 2024; Zheng and Zhang, 2024).

**Figure 3:**
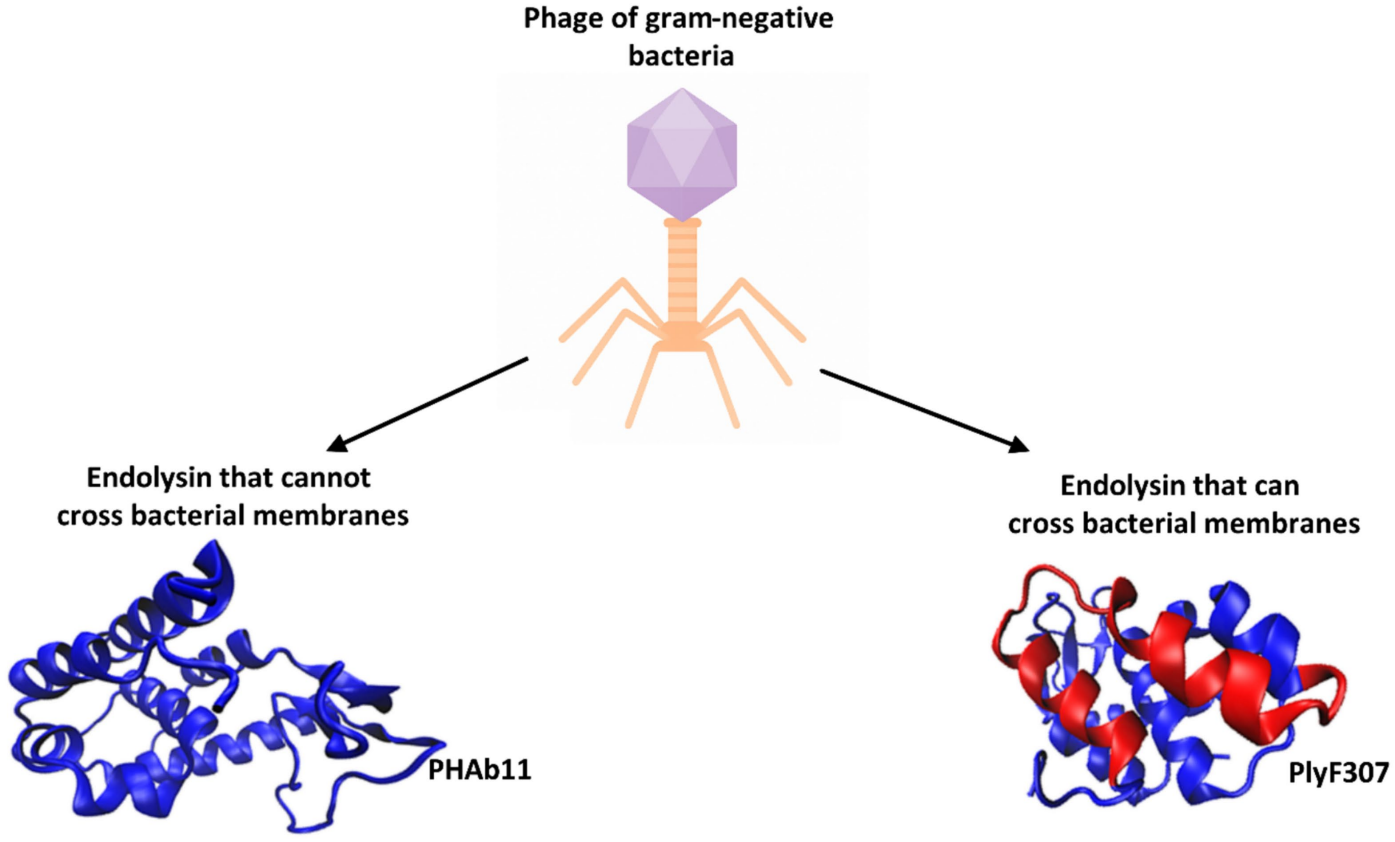
Endolysins derived from bacteriophages active against gram-negative bacteria are classified into two groups. An example of an endolysin belonging to the first group is PHAb11, which contains a single EAD domain with lysozyme activity (PDB code: 9KBS, Hu, 2024). An example of an endolysin capable of crossing bacterial membranes is PlyF307 (its structure was predicted using the I-TASSER program) (Yang and Zhang, 2015). The helical fragment isolated as an independent antibacterial peptide is marked in red.

Endolysins are fast-acting, potent, and inactive against eukaryotic cells. Combining phage lysins with antibiotics may enhance infection treatment efficacy compared to antibiotics alone. Bacterial resistance to lysins is considered unlikely because lysins target critical PG sites essential for bacterial viability (Ghose and Euler, 2020; Gondil et al., 2020; Gontijo et al., 2021; Rahman et al., 2021; Vázquez et al., 2021; Khan et al., 2023, 2024; Sisson et al., 2024b; Zheng and Zhang, 2024). Phage endolysin activity can induce lysis from the outside due to high intracellular osmotic pressure, which serves as a basis for exploring purified phage endolysins as antimicrobial agents against gram-positive pathogens (Liu H. et al., 2023; Vander Elst, 2024). However, in gram-negative bacteria, the outer membrane often prevents endolysin access, requiring surfactants or additional mechanisms for translocation. Nonetheless, some bacteriophage-derived endolysins exhibit antibacterial activity against gram-negative pathogens. In this review, endolysins were classified into two groups (Figure 3):

1. Endolysins that cannot penetrate the gram-negative bacterial cell wall.
2. Endolysins that can penetrate the gram-negative bacterial cell wall.

In the following sections, existing methods for enhancing the activity of endolysins in the first group and present strategies proposed by various research groups for designing novel, membrane-active peptide sequences utilizing endolysins from the second group.

## 3 The use and limitations of endolysins in antibacterial therapies against gram-negative bacteria

Endolysins were initially considered ineffective against gram-negative bacteria due to the protective outer membrane of these organisms. However, various strategies had been developed to enhance their efficacy, including the use of outer membrane permeabilizers, fusion with membrane-active peptides, nanotechnology-based delivery methods, and liposome encapsulation. This review focuses on the key approaches for utilizing endolysins in therapies targeting gram-negative bacteria, specifically, it highlights methods involving membrane-permeabilizing agents and strategies that enhance endolysin activity through fusion with membrane-degrading peptides.

### 3.1 Combination of endolysins with outer membrane permeabilizes

**Figure 4:**
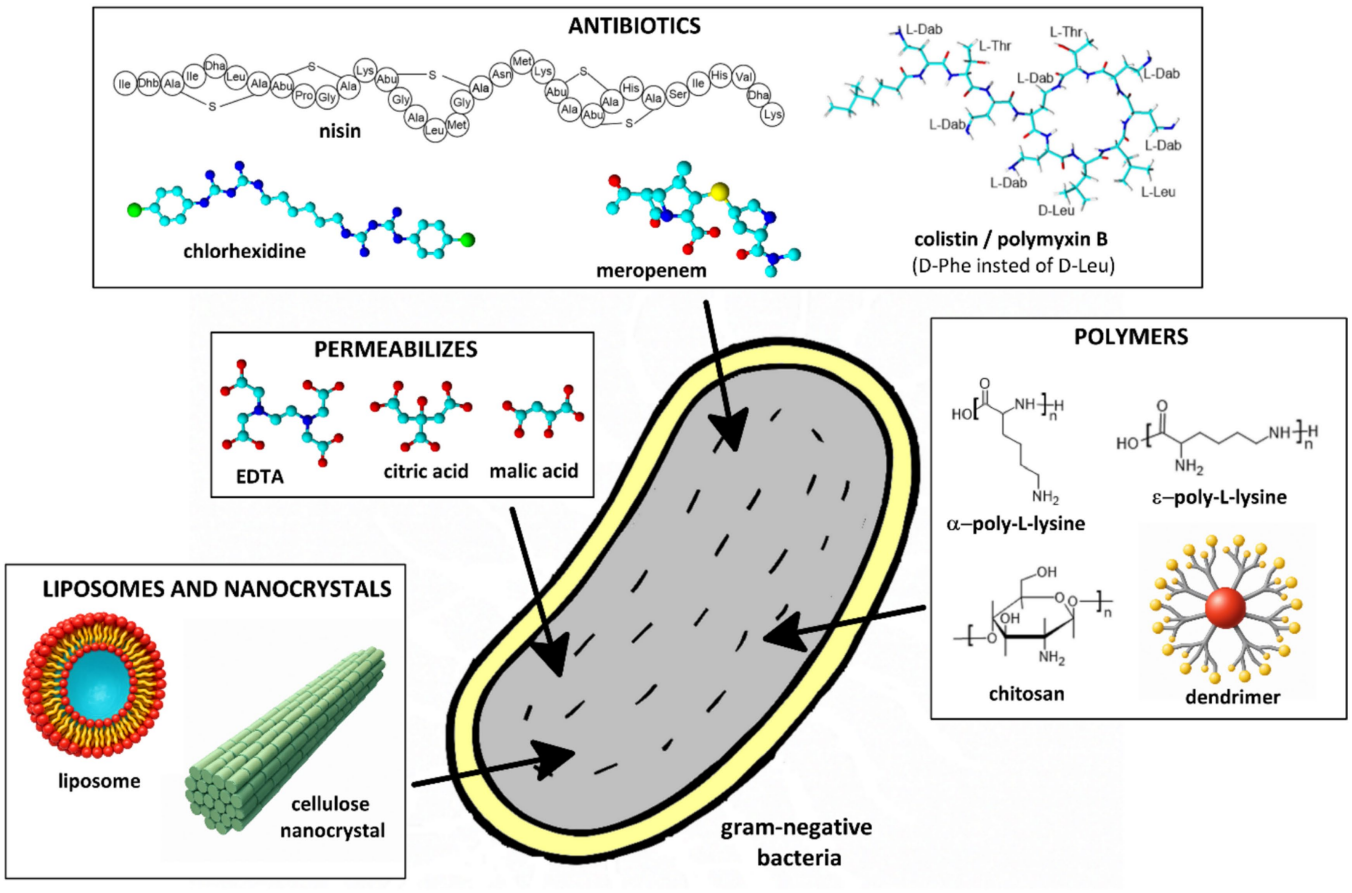
Schematic representation of selected outer membrane permeabilizers used to enhance the efficacy of endolysins against gram-negative bacteria.

Combining endolysins with outer membrane permeabilizers is a promising strategy to enhance their activity against gram-negative bacteria (Figure 4; Table 1). These permeabilizers disrupt the outer membrane by chelating divalent cations, making the peptidoglycan layer accessible to endolysins. The main approaches utilizing permeabilizers in combination with endolysins are outlined below.

**Table 1:** Examples of combinations of endolysins with outer membrane permeabilizers.

Endolysin	Permeabilizers	Antimicrobial activity spectrum	References
OBPgp279 fused with cecropin A	EDTA and citric acid	*A. baumannii* and *P. aeruginosa*	Yang et al. (2015)
OBPgp279, PVP-SE1gp146, 201φ2-1gp229	EDTA	*P. aeruginosa*, *E. coli*, *S. enterica*	Walmagh et al. (2012)
BcepC6gp22, P2gp09, PsP3gp10, K11gp3.5, KP32gp15	EDTA	*P. aeruginosa*	Walmagh et al. (2013)
LysPN09	EDTA	*Pseudomonas syringae pv. actinidiae*	Ni et al. (2021)
LysMK34 and LysMK34 fused with cecropin A	EDTA	*A. baumannii*	Abdelkader et al. (2022)
KZ144 and fused with SMAP-29	EDTA	*P. aeruginosa*	Briers et al. (2014a)
		*A. baumannii*	Defraine et al. (2016)
LysABP-01	Colistin	*A. baumannii*	Thummeepak et al. (2016)
ElyA1	Colistin	*A. baumannii*, *P. aeruginosa, K. pneumoniae*	Blasco et al. (2020)
lysAB-vT2 and lysAB-vT2-fusion	Colistin, polymyxin B, and CuCl_2_	*A. baumannii*, *K. pneumoniae*, *E. coli*	Sitthisak et al. (2023)
LysAB2	Colistin	*A. baumannii*, *K. pneumoniae*, *E. coli*, *P. aeruginosa*, *S. enterica*	Zhang P. et al. (2024)
rEnd2 OAM-rEnd2	Colistin	*P. aeruginosa*	Oliveira et al. (2024)
T7L T4L	Colistin, nisin, and polymyxin B	*P. aeruginosa* *S. aureus*	Tyagi et al. (2024)
rLysJNwz	EDTA, polymyxin B, citric acid, malic acid	*S. enterica*	Shen et al. (2023)
EndoT5	Polymyxin B, chlorhexidine, poly-L-lysine	*E. coli*	Shavrina et al. (2016)
LysPB32	Depolymerase DpolP22	*S. enterica*	Kim et al. (2023)
LysE	EDTA, colistin	*A. hydrophila*	Baliga et al. (2023)
Lys68	EDTA, citric acid, malic acid	*S. enterica*	Oliveira et al. (2014)
ABgp46	Citric acid and malic acid	*A. baumannii*, *P. aeruginosa*, and *S. enterica*	Oliveira et al. (2016)
Ts2631	EDTA, citric acid, malic acid	*A. baumannii*, *P. aeruginosa*, *Enterobacteriaceae* family	Plotka et al. (2015, 2019)
PlyPa03	Citric acid	*P. syringae*	Sisson et al. (2024a)
CF-370	Meropenem	*P. aeruginosa*	Sauve et al. (2024)
EC340	Meropenem, colistin	*E. coli*	Hong et al. (2022)
XFII	Chitosan	*E. coli*	Zhang et al. (2022)
Lysqdvp001	ε-poly-lysine (ε-PL)	*V. parahaemolyticus*	Ning H. Q. et al. (2021)
EL188	EDTA, citric acid, poly-L-lysine, polymyxin B nonapeptide	*P. aeruginosa*	Briers et al. (2011)
KP27	Dendrimers	*P. aeruginosa*	Ciepluch et al. (2019)
KP27	Cationic carbosilane (CBS) dendrimers	*P. aeruginosa*	Quintana-Sanchez et al. (2022)
BSP16Lys	Liposomes	*E. coli*, *S. enterica*	Bai et al. (2019)
Pa7, Pa119	Liposomes	*P. aeruginosa*	Morais et al. (2022)
Lysqdvp001	Liposomes	*V. parahaemolyticus*	Ning et al. (2023)
T4	Cellulose nanocrystals	*E. coli* and *Pseudomonas mendocina*	Abouhmad et al. (2017)

#### 3.1.1 Use of endolysins with chemical permeabilizes

Studies show that endolysins combined with permeabilizers such as ethylenediaminetetra acetic acid (EDTA) or organic acids lead to a notable increase in bacterial cell lysis (Figure 4). The combination of endolysin LysE and EDTA demonstrated a biofilm-preventive effect against *Aeromonas hydrophila*. EDTA enhanced the access of LysE to the bacterial cell wall, leading to a one-log reduction in *A. hydrophila* planktonic cells (Baliga et al., 2023). The endolysin SPN9CC, when used with EDTA, exhibited a significant bactericidal effect against *E. coli.* A combination of 300 μg/mL of endolysin with 1 mM EDTA resulted in a four-log reduction in bacterial counts—double the reduction observed with SPN9CC alone. EDTA alone did not significantly affect bacterial viability, indicating that its role was primarily to enhance endolysin access by disrupting the outer membrane (Lim et al., 2014). The endolysins OBPgp279, PVP-SE1gp146, 201φ2-1gp229, when combined with EDTA, showed increased antibacterial activity against *P. aeruginosa*. This effect was attributed to EDTA’s ability to chelate divalent cations, destabilizing the outer membrane and allowing endolysin access (Walmagh et al., 2012). The combination of two endolysins (PsP3gp10 and K11gp3.5) with EDTA led to a 2.62-log reduction in *P. aeruginosa* PAO1 viability, demonstrating an effective strategy for enhancing endolysin-based antibacterial approaches. Briers et al. explored explored the use of outer membrane permeabilizers such as EDTA, citric acid, poly-L-lysine, and polymyxin B nonapeptide to enhance the antibacterial activity of the bacteriophage endolysin EL188 against *P. aeruginosa*. Among these, EDTA was found to be the most effective, significantly increasing bacterial permeability and enabling EL188 to reduce *P. aeruginosa* viability by up to 4 log units within 30 min. The study highlighted a strain-dependent effect, with higher-resistance strains requiring greater concentrations of EDTA for efficient permeabilization (Briers et al., 2011).

In addition to EDTA, other permeabilizers such as organic acids have been investigated for their ability to disrupt the outer membrane and potentiate the activity of endolysins (Oliveira et al., 2014, 2016; Plotka et al., 2015, 2019; Shen et al., 2023). Among them, citric acid and malic acid (Figure 4) have shown promising effects due to their capacity to acidify the microenvironment and destabilize the lipopolysaccharide (LPS) layer without relying on metal ion chelation. Unlike EDTA, which functions by sequestering divalent cations (Mg^2+^, Ca^2+^) and disrupting ionic bridges in the LPS structure, organic acids exert their activity primarily by reducing the pH and protonating phosphate groups within the outer membrane, leading to membrane destabilization. Oliveira et al. investigated the antibacterial potential of the thermostable Lys68 endolysin derived from an *S. enterica* phage. The study focused on how weak acids (EDTA, citric acid, malic acid) act as permeabilizers, enhancing the bactericidal activity of Lys68 against gram-negative pathogens. Weak acids disrupt the outer membrane, allowing Lys68 to hydrolyze the bacterial cell wall, leading to rapid bacterial lysis. Without permeabilizers, Lys68 alone showed limited activity, but in combination, it exhibited significantly enhanced bactericidal effects against *S. enterica*, *E. coli*, and *K. pneumoniae*. The permeabilizers also contributed to biofilm disruption, increasing overall antimicrobial efficacy. The study confirmed that Lys68 remains thermostable, retaining its activity under various temperature conditions, which could be advantageous for industrial and medical applications (Oliveira et al., 2014). The same authors (Oliveira et al., 2016) demonstrate that organic acids played a crucial role in enhancing the antibacterial efficacy of the endolysin ABgp46 against gram-negative bacteria. Citric acid and malic acid were tested as outer membrane permeabilizers, allowing ABgp46 to reach and degrade the bacterial cell wall, leading to effective bacterial lysis. The same permeabilizer improved the lytic activity of Ts2631 endolysin against MDR gram-negative pathogens, including *P. aeruginosa* and *K. pneumoniae* (Plotka et al., 2015, 2019). Other authors investigated the potential of combining a phage-derived endolysin with citric acid to enhance antibacterial efficacy against *Pseudomonas syringae pv. actinidiae* (Psa), an important plant pathogen affecting kiwifruit. The research focuses on the endolysin PlyPa03, which, on its own, had limited activity against *P. syringae* due to the protective outer membrane of gram-negative bacteria. However, the addition of citric acid as a permeabilizer destabilized the bacterial outer membrane, allowing PlyPa03 to access and degrade the peptidoglycan layer. Experimental results demonstrate that the combination significantly improves bacterial lysis compared to either agent alone, suggesting a promising biocontrol strategy for managing Psa infections in kiwifruit orchards (Sisson et al., 2024a).

Recent studies have demonstrated that combining citric or malic acid with phage-derived endolysins can significantly enhance bactericidal activity against *Salmonella* spp. under mildly acidic conditions (pH 5.0–6.0) (Oliveira et al., 2014, 2016). These combinations were particularly effective in reducing bacterial viability in food preservation and clinical isolate contexts, and represent an attractive alternative for situations where chelating agents such as EDTA are not desirable. Importantly, organic acids are generally regarded as safe and are already used in food and pharmaceutical formulations, which supports their potential in translational applications involving endolysin-based antimicrobials.

The use of chemical permeabilizers such as EDTA and weak acids improves the efficacy of endolysins against gram-negative bacteria. These combinations enhance bacterial cell wall penetration, leading to increased bactericidal activity, and biofilm disruption (Walmagh et al., 2012, 2013; Briers et al., 2014a; Lim et al., 2014; Yang et al., 2015; Defraine et al., 2016; Ni et al., 2021; Abdelkader et al., 2022; Baliga et al., 2023).

#### 3.1.2 Combining endolysins with antibiotics

Combining endolysins with antibiotics that disrupt the outer membrane had also been shown to improve antibacterial activity (Figure 4). For instance, Thummeepak et al. investigated the antibacterial activity of the endolysin LysABP-01, derived from the *A. baumannii* bacteriophage ØABP-01. Their study demonstrated that LysABP-01 exhibited significant lytic activity against *A. baumannii*, particularly when combined with colistin—a last-resort antibiotic. Colistin disrupts the outer membrane, enhancing endolysin efficacy. The synergistic effect significantly increased bacterial killing, suggesting a promising strategy for treating multidrug-resistant *A. baumannii* infections (Thummeepak et al., 2016). Similarly, Blasco et al. tested the efficacy of combining colistin with the endolysins ElyA1 and ElyA2 against multidrug-resistant bacterial strains. Their study demonstrated enhanced antibacterial activity both *in vitro* and *in vivo* when endolysins were used in combination with colistin (Blasco et al., 2020). Another example involves Sitthisak et al. who developed recombinant endolysin LysAB-vT2 and its hydrophobic fusion variant LysAB-vT2-fusion. These endolysins exhibited a synergistic effect with polymyxin antibiotics (colistin, polymyxin B). The hydrophobic fusion variant improved bacterial lysis by enhancing the ability of the endolysin to interact with and disrupt the bacterial cell wall. The combination with polymyxins further potentiated their antimicrobial activity, providing a robust approach against drug-resistant gram-negative bacteria (Sitthisak et al., 2023). The research demonstrated that when used together, colistin and endolysins exhibit a synergistic effect, significantly improving bacterial killing compared to either agent alone. This combination therapy offers a promising strategy to combat infections caused by multidrug-resistant gram-negative bacteria (Zhang P. et al., 2024). The study presented by Oliveira et al. (2024) investigated a novel endolysin derived from the *P. aeruginosa* bacteriophage vB_PaeM_USP2. Biochemical assays demonstrated that rEnd2 possesses peptidoglycan-degrading activity, effectively targeting the bacterial cell wall. When combined with colistin, rEnd2 exhibited a synergistic antibacterial effect against *P. aeruginosa* (Oliveira et al., 2024). Tyagi et al. analyzed the interaction between bacteriophage endolysins (T7L, T4L) and antimicrobial peptides (AMPs) such as colistin, nisin, and polymyxin B to combat bacterial biofilms. Their study demonstrated that the combination provides a synergistic effect, significantly enhancing the eradication of bacterial biofilms compared to using either agent alone. This approach could be especially valuable for treating persistent bacterial infections protected by biofilms (Tyagi et al., 2024). The combination of endolysins and permeabilizers also presents a promising antibacterial strategy for food safety applications, helping to control *S. enterica* contamination in food products (Shen et al., 2023). The study (Shavrina et al., 2016) demonstrated that the bacteriophage T5 endolysin (EndoT5) showed significantly enhanced antibacterial activity against *E. coli* when combined with membrane-permeabilizing agents. The most effective permeabilizers included polymyxin B and chlorhexidine, which reduced bacterial counts by five orders of magnitude. Poly-L-lysine also contributed to a four-order reduction in CFUs (colony-forming units). The study highlighted a strong synergistic effect between EndoT5 and these agents, enabling efficient bacterial lysis (Shavrina et al., 2016).

A very interesting study prepared by Kim et al. (2023) explores the synergistic antibacterial activity of endolysin and depolymerase in combating *S. enterica* biofilms. While depolymerases break down biofilm exopolysaccharides, making bacteria more vulnerable to antimicrobial agents, the endolysins degrade the peptidoglycan layer in bacterial cells. The combination of phage-derived endolysin and depolymerase significantly enhanced biofilm disruption and bacterial killing compared to either enzyme alone. The dual-enzyme approach effectively reduced biofilm biomass and increased the susceptibility of biofilm-embedded *S. typhimurium* cells to lysis. This strategy presents a promising alternative to conventional antibiotics for controlling persistent *S. enterica* infections, especially in environments promoting the bacterial biofilm formation (Kim et al., 2023).

Sauve et al. investigated the efficacy of CF-370, an engineered lysin, against multidrug-resistant gram-negative bacteria, particularly *P. aeruginosa*. CF-370 demonstrated potent antimicrobial activity with minimum inhibitory concentrations (MICs) for *P. aeruginosa* at 0.5 μg/mL. Notably, CF-370 exhibited bactericidal properties, effectiveness in human serum, low resistance development, anti-biofilm activity, and synergistic effects when combined with antibiotics. In a rabbit acute pneumonia model, combining CF-370 with meropenem significantly improved bacterial clearance from lung tissues compared to either agent alone, suggesting a promising therapeutic approach for antibiotic-resistant gram-negative bacteria (Sauve et al., 2024). Hong et al. demonstrated that combining EC340 with antibiotics such as colistin or meropenem significantly increased bactericidal effects against *E. coli* and other resistant gram-negative pathogens. The combinations allowed for lower antibiotic concentrations while maintaining or improving antimicrobial efficacy, which was crucial for preventing antibiotic resistance development. Additionally, the EC340-antibiotic combination effectively disrupted bacterial biofilms, which were often resistant to conventional treatments (Hong et al., 2022).

Baliga et al. explored alternative antimicrobial strategies against multidrug-resistant *Aeromonas hydrophila*, a gram-negative pathogen found in freshwater environments. The purified recombinant LysE protein showed substantial antimicrobial activity against *A. hydrophila*, particularly when combined with membrane-permeabilizing agents such as EDTA. LysE also reduced biofilm formation and exhibited synergistic effects with low concentrations of colistin. Importantly, cytotoxicity assays using *Channa striatus* kidney cell lines indicated that LysE was non-toxic to animal cells, highlighting its potential as an effective antimicrobial agent in aquaculture, food safety, and healthcare applications (Baliga et al., 2023).

#### 3.1.3 Combination of endolysins with polymers

Polymer-based approaches, including the use of permeabilizers, dendrimers, liposomes, and enzyme immobilization, have been explored to enhance the efficacy of endolysins against gram-negative bacteria (Figure 4). Zhang et al. characterized *S. enterica* endolysin XFII, produced recombinantly in *E. coli*, evaluating its lytic activity, stability, and antibacterial efficacy. Additionally, the researchers explored combining XFII with chitosan, a natural antimicrobial agent, to enhance bacterial lysis. The combination of XFII and chitosan significantly improved antibacterial activity, offering a promising solution for treating gram-negative infections (Zhang et al., 2022). Another study investigated the impact of combining endolysin Lysqdvp001 with a permeabilizer *ε*-poly-lysine to enhance its antibacterial activity against *Vibrio parahaemolyticus* and its biofilms. The permeabilizer facilitated Lysqdvp001 penetration through the bacterial outer membrane, significantly improving its lytic efficiency and disrupting biofilms resistant to conventional antimicrobial agents (Ning H. Q. et al., 2021).

Dendrimers are highly branched, nano-sized macromolecules with a well-defined, symmetrical structure. Their unique architecture allows them to be monodisperse, meaning they have uniform size and structure, unlike conventional polymers. Due to their high functional group density, dendrimers exhibit exceptional properties such as high solubility, low toxicity, and the ability to form multivalent interactions. These characteristics make them particularly useful in biomedical applications, including drug delivery, gene therapy, diagnostic imaging, and antimicrobial treatments (Abbasi et al., 2014). The study Ciepluch et al. (2019) investigated the potential of combining phage-derived endolysins with cationic dendrimers to combat *P. aeruginosa*, a gram-negative bacterium notorious for antibiotic resistance. Cationic dendrimers, which carry a positive charge, can disrupt the bacterial outer membrane, enhancing the accessibility and activity of endolysins. Their study demonstrated that a specific phage endolysin combined with poly(propyleneimine) dendrimers resulted in significantly improved bacterial lysis compared to the endolysin alone. This suggests that cationic dendrimers aid in penetrating the outer membrane, enhancing the overall antibacterial efficacy of endolysins (Ciepluch et al., 2019). Quintana-Sanchez et al. studied PEGylated carbosilane dendrimers, which exhibited direct antibacterial activity against *P. aeruginosa* by disrupting bacterial membranes. When combined with a phage-derived endolysin, the antibacterial effect was significantly enhanced, suggesting a synergistic interaction. The study demonstrated that dendrimers facilitated better access of endolysins to the bacterial peptidoglycan layer by compromising the outer membrane. This combination therapy was particularly effective against antibiotic-resistant strains, highlighting its potential as an alternative antimicrobial strategy (Quintana-Sanchez et al., 2022).

#### 3.1.4 Liposomal and nanotechnology-based delivery systems

Another innovative approach explored the use of liposomes as delivery systems for endolysins to enhance their efficacy against gram-negative bacteria (Gondil and Chhibber, 2021). The researchers encapsulated endolysins within liposomes, which serve as carriers to facilitate their transport and release at the bacterial cell wall. The study involved formulating and characterizing cationic liposomes containing dipalmitoylphosphatidylcholine (DPPC), cholesterol, and hexadecylamine, encapsulation BSP16Lys endolysin efficiency, and antimicrobial activity tests. The results demonstrated that liposome-encapsulated endolysins exhibited significantly improved antibacterial effects compared to free endolysins, particularly against multidrug-resistant gram-negative strains (Bai et al., 2019). Other authors, Morais et al. encapsulated newly identified prophage lysins (Pa7 and Pa119) within DMPC (dimirystoilofosfatydylocholina):DOPE (dioleoyl phosphatidylethanolamine):CHEMS (cholesteryl hemisuccinate) (4:4:2 molar ratio) liposomes to enhance their antibacterial efficacy against *P. aeruginosa*. The study demonstrated improved stability, bioavailability, and penetration of bacterial defenses. Evaluations both *in vitro* and in a *P. aeruginosa* infection model confirmed that liposome-encapsulated lysins exhibited enhanced antibacterial effects compared to free lysins (Morais et al., 2022). Encapsulation of phage lysin Lysqdvp001 in liposomes to enhance its stability and antibacterial activity against seafood-related pathogens was also tested. The liposomal formulation effectively inhibited the growth of *Vibrio parahaemolyticus* in live clams, reducing the risk of spoilage. The treatment showed no significant impact on the quality or viability of the clams, making it a promising alternative to traditional preservatives (Ning et al., 2023).

The immobilization of lysozymes (hen egg white lysozyme and T4 lysozyme) onto positively charged cellulose nanocrystals (CNCs) was used to enhance their antibacterial activity and stability. Immobilization onto CNCs protected lysozymes from degradation and extended their functional lifespan. The modified lysozymes showed stronger antibacterial effects against gram-positive and gram-negative bacteria compared to free enzymes. The immobilized enzymes provided a gradual and prolonged antibacterial effect, making them more effective in long-term applications (Abouhmad et al., 2017).

### 3.2 Modifications of endolysins for improving outer membrane penetration

Genetic engineering of endolysins to enhance their hydrophobic properties and positive charge has become a crucial strategy for improving their efficacy against gram-negative bacteria. These modifications help endolysins overcome the barrier posed by the negatively charged LPS in the outer membrane.

#### 3.2.1 Fusion of endolysins with antimicrobial peptides

**Figure 5:**
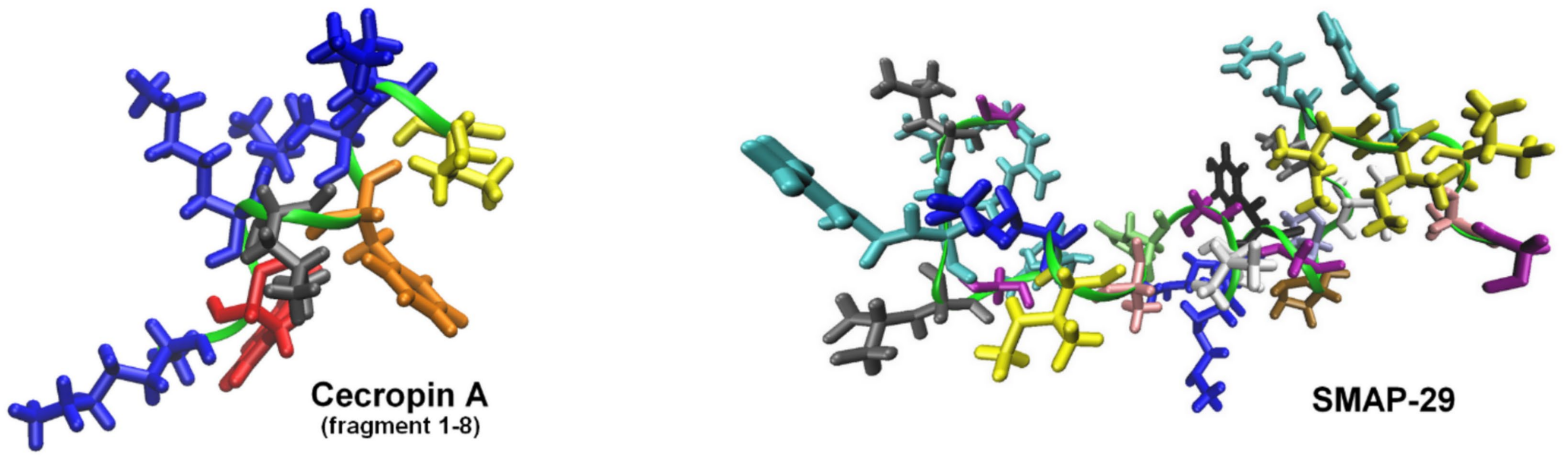
Structures of representative peptides fused with endolysins to enhance their antibacterial activity against gram-negative bacteria. The models of cecropin A (fragment 1-8) and SMAP-29 peptides were visualized based on the solution NMR structure with PDB codes: 1DYJ (Oh et al., 1999) and 1FRY (Tack et al., 2002), respectively. Peptides form helices marked as green ribbon, with the Lys side-chains shown in blue, Trp in red, Leu in gray, Phe in orange, Ile in yellow, Ala in pink, Arg in cyan, Gly in purple, His in lime, Pro in ochre, Thr in iceblue, Tyr in black, and Val in white. The figures were prepared with VMD (Humphrey et al., 1996).

**Figure 6:**
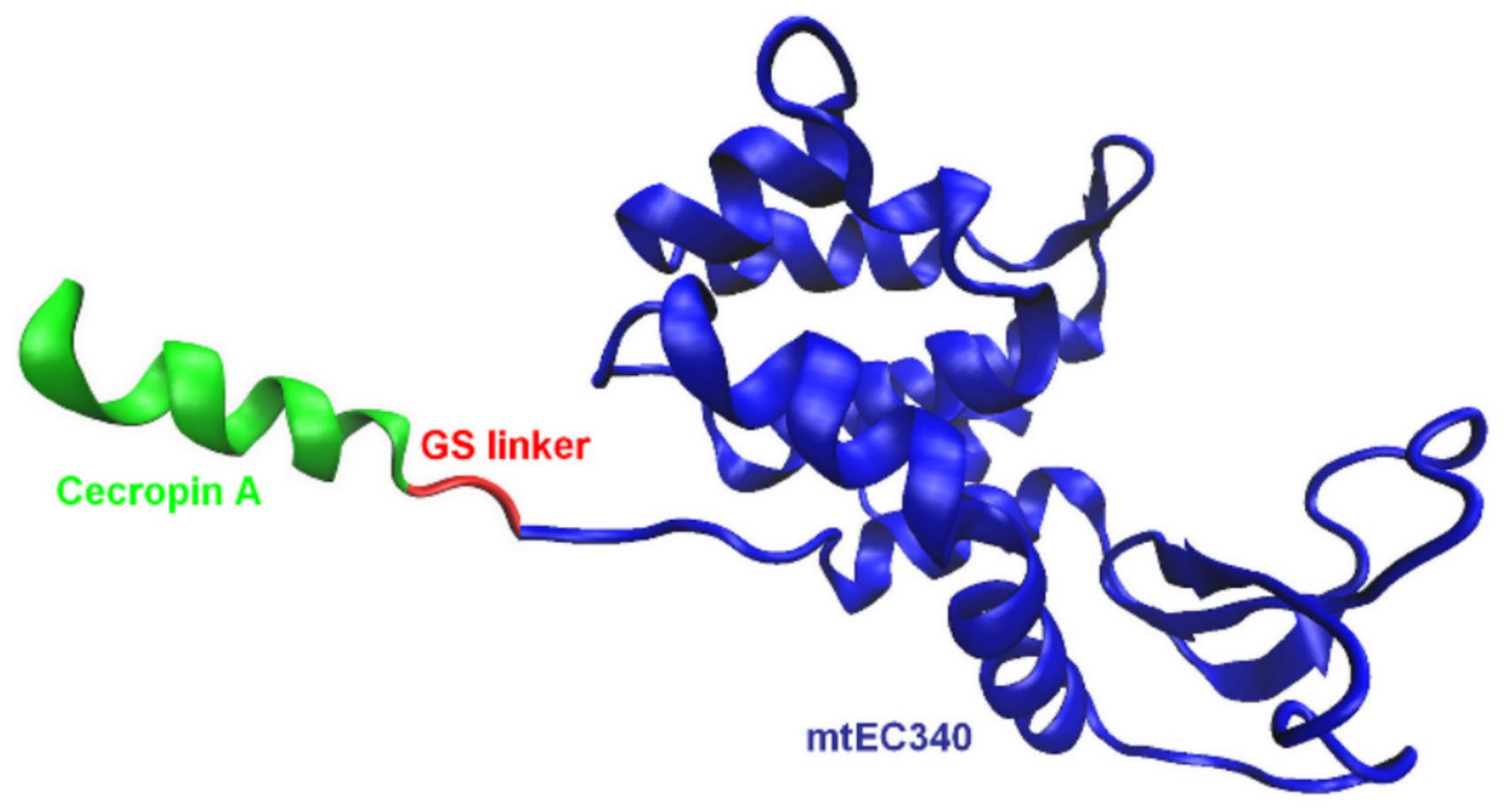
Schematic representation of an example of endolysin fusion with a peptide: LNT113 (Cho et al., 2024). The artilysin is composed of the Cecropin A peptide fused to the mtEC340 endolysin. The model of the mtEC340 endolysin was visualized based on the X-ray crystallography structure, PDB code: 8HP8 (Wang et al., 2023). The figure was prepared using VMD software (Humphrey et al., 1996).

One of the most effective approaches involves fusing endolysins with membrane-active peptides to facilitate outer membrane penetration and enhance bactericidal activity (Table 2). A notable example is Artilysins^®^, developed by Lysando AG, which are engineered fusion proteins combining endolysins with antimicrobial or membrane-penetrating peptides (Schirmeier et al., 2018; Lysando, 2025). These hybrid molecules disrupt the outer membrane of gram-negative bacteria, allowing the catalytic domain of the endolysin to access and degrade the PG layer (Carratalá et al., 2023). Examples of such constructs include the fusion of the cecropin A peptide (Figure 5) with OBPgp279W (Yang et al., 2015), AbEndolysin (Islam et al., 2022), and the LysMK34 endolysin (Abdelkader et al., 2022). In a study by Lim et al., researchers engineered the ST01 endolysin fused with cecropin A to target multidrug-resistant *A. baumannii*. This construct exhibited potent antimicrobial activity against clinical isolates. The fusion effectively disrupted bacterial membranes, leading to rapid cell death and significantly reduced bacterial counts in a mouse bacteremia model, highlighting its therapeutic potential (Lim et al., 2022a). Similarly, Jeong et al. (2023) characterized three distinct endolysins (Lys10-24(13), LysPBEC30, and LysPBEC56) and evaluated their bactericidal efficiency and structural properties. Their research demonstrated that fusing these endolysins with cecropin A enhanced their outer membrane penetration and antibacterial activity against *E. coli*, *P. aeruginosa*, *A. baumannii*, and *K. pneumoniae* (Jeong et al., 2023). Cho and You further demonstrated that fusing cecropin A to the endolysin mtEC340 significantly improved its interaction with LPS and increased its bactericidal activity against *E. coli* model (Figure 6). Their study emphasized that the cecropin A fusion accelerated bacterial membrane disruption, facilitating rapid cell death. This strategy also proved effective against bacterial strains with defects in their core oligosaccharides, underscoring the importance of LPS interaction in enhancing endolysin activity (Cho et al., 2024). LysAB2, derived from *A. baumannii* phage, was fused with the cecropin A (1-8) fragment, improving its penetration through the outer membrane and increasing its activity against multidrug-resistant *A. baumannii* (Chen et al., 2021).

Other studies described a conjugation of LysPA26 (Wang et al., 2021) and KZ144 (Briers et al., 2014a; Defraine et al., 2016) with antimicrobial peptide SMAP-29 (Figure 5). Art-085 and Art-175, derived from KZ144 fused with SMAP-29, exhibited enhanced bactericidal efficacy against pathogens like *P. aeruginosa*, *E. coli* (Briers et al., 2014a), and *A. baumannii* (Defraine et al., 2016). In another study, Antonova et al. explored how fusing different cationic and amphipathic peptides to the endolysin LysECD7 improved its bactericidal activity against gram-negative bacteria. This research demonstrated that specific peptide tags significantly enhanced the enzyme’s lytic efficiency (Antonova et al., 2020; Vasina et al., 2024). Sitthisak et al. further optimized endolysins by adding hydrophobic amino acids to the C-terminus of LysAB-vT2, increasing its lytic activity. These hydrophobic fusion constructs exhibited enhanced efficacy when combined with colistin and polymyxin B (Sitthisak et al., 2023). A particularly innovative fusion, Tha-PA90, developed by Lim et al. combined the antimicrobial peptide thanatin with the endolysin PA90. This fusion exhibited strong lytic activity against *A. baumannii*, including antibiotic-resistant strains, and effectively disrupted biofilms. *In vivo* studies using a mouse infection model confirmed that Tha-PA90 significantly reduced bacterial loads and improved survival rates, making it a promising alternative for treating *A. baumannii* infections (Lim et al., 2024). A polycationic nonapeptide (PCNP) had also been used as a carrier to deliver endolysins into gram-negative bacterial cells. One study demonstrated that PCNP effectively addressed a broad range of MDR gram-negative pathogens, making it a versatile tool for combating various bacterial infections (Briers et al., 2014b). Another study focused on *Helicobacter pylori*, emphasizing its role in gastrointestinal diseases (Xu et al., 2021).

Ma et al. engineered Lysep3 by inserting cationic peptides into its C-terminal region, increasing its interaction with the bacterial outer membrane and improving its antimicrobial activity (Ma et al., 2017). The same cationic peptides were fused to the Lysqdvp001 endolysin, significantly enhancing antimicrobial activity against *V. parahaemolyticus* strains (Ning H. et al., 2021). Similar results were achieved by Yan et al. who introduced hydrophobic amino acids to Lysep3, increasing its lytic efficacy under specific pH conditions (Yan et al., 2019). Another innovative approach involved combining endolysins with sensitizer peptides to improve their ability to penetrate the outer membrane. Son, Kim, and Ryu engineered Lys1S-L9P by fusing Lys1S with the sensitizer peptide KL-L9P, with enhanced bactericidal activity against MDR *E. coli*, *P. aeruginosa*, and *A. baumannii* (Son et al., 2023). Lim et al. fused the PA90 endolysin with a cell-penetrating peptide (CPP), DS4.3, demonstrated efficient bacterial eradication, biofilm disruption, and reduced cytotoxicity *in vitro* and *in vivo* (Lim et al., 2022b). Li et al. improved the antibacterial efficacy of LysECD7 by fusing it with LPS-interacting peptides and modifying it with fatty acid derivatization. These modifications significantly enhanced bacterial killing and infection clearance (Li et al., 2024). The novel recombinant endolysin (rEnd2), derived from *P. aeruginosa* phage vB_PaeM_USP2, particularly when combined with colistin. Further conjugation of rEnd2 with polycationic-polymer nanoparticles improved its stability and antimicrobial activity, suggesting promising potential for treating MDR bacterial infections (Oliveira et al., 2024).

In the study prepared by Siu et al. researchers constructed a chimeric endolysin library by inserting an oligonucleotide sequence of 20 repeated NNK codons upstream of the endolysin gene Bp7e. This library was expressed in *E. coli* and screened for variants exhibiting extracellular antibacterial activity. One engineered endolysin, named Art-Bp7e6, demonstrated significant bactericidal effects against *E. coli*. Further analysis revealed that the chimeric peptide depolarized the bacterial cell envelope, increased cell permeability, and facilitated enzyme access to degrade peptidoglycan, providing a novel platform for engineering and screening highly active endolysins (Sui et al., 2023). Other researchers engineered endolysins derived from a *K. pneumoniae* phage, fusing them with peptides ApoE23 and COG133 to create chimeric proteins. These engineered endolysins were tested *in vitro* against ESKAPEE pathogens (*Enterococcus faecium*, *S. aureus*, *K. pneumoniae*, *A. baumannii*, *P. aeruginosa*, *Enterobacter* spp., and *E. coli*). The results demonstrated that the chimeric endolysin ApoE23-Kp84B (CHU-1) achieved a reduction growth of *A. baumannii*, *E. faecalis*, and *K. pneumoniae*. Similarly, COG133-Kp84B (CHU-2) exhibited significant efficacy against *S. aureus* (Chen et al., 2024).

These studies highlight the substantial progress in engineering endolysins through genetic modifications and peptide fusions. These advancements enhance their ability to penetrate the outer membrane of gram-negative bacteria, offering promising therapeutic options against MDR pathogens (Table 2).

**Table 2:** Examples of fusions of endolysins with peptides.

Artilysin	Endolysin	Peptide	Sequence	Peptide orientation	Linker	Antimicrobial activity spectrum	References
PlyA	OBPgp279W	Cecropin A (fragment 1-8)	KWKLFKKI	N-terminal	(GGGGS)2	*A. baumannii*, *P. aeruginosa*	Yang et al. (2015)
eAbEndolysin	AbEndolysin	Cecropin A	KWKLFKKIEKVGQNIRDGIIKAGPAVAVVGQATQIAK	N-terminal	–	*A. baumannii*	Islam et al. (2022)
eLysMK34	LysMK34	Cecropin A	KWKLFKKIEKVGQNIRDGIIKAGPAVAVVGQATQIAK	N-terminal	–	*A. baumannii*	Abdelkader et al. (2022)
CecA::ST01	ST01	Cecropin A	KWKLFKKIEKVGQNIRDGIIKAGPAVAVVGQATQIAK	N-terminal	–	*P. aeruginosa*, *K. pneumoniae*, *A. baumannii*, *E. coli*, and *E. cloacae*	Lim et al. (2022a)
CecA-Lys10-24(13) CecA-LysPBEC30 CecA-LysPBEC56	Lys10-24(13) LysPBEC30 LysPBEC56	Cecropin A	KWKLFKKIEKVGQNIRDGIIKAGPAVAVVGQATQIAK	N-terminal	(GSGSGS)_3_	*E. coli*, *P. aeruginosa*, *A. baumannii*, *K. pneumonia*	Jeong et al. (2023)
LNT113	mtEC340	Cecropin A	KWKLFKKIEKVGQNIRDGIIKAGPAVAVVGQATQIAK	N-terminal	GS linker	*E. coli*	Cho et al. (2024)
LysAB2-KWK	LysAB2	Cecropin A (fragment 1-8)	KWKLFKKI	C-terminal	GGSGG	*A. baumannii*, *E. coli*, *A. baumannii* biofilm	Chen et al. (2021)
AL-3AA, AL-9AA, AL-15AA	LysPA26	SMAP-29	RGLRRLGRKIAHGVKKYGPTVLRIIRIAG	N-terminal	GSA (GSA)_3_ (GGGGS)_3_	*P. aeruginosa*, *K. pneumoniae*, *E. coli*, and *P. aeruginosa* biofilms	Wang et al. (2021)
Art-085 and Art-175	KZ144	SMAP-29	RGLRRLGRKIAHGVKKYGPTVLRIIRIAG	N-terminal	*–*	*P. aeruginosa*, *E. coli*	Briers et al. (2014a)
						*A. baumannii*	Defraine et al. (2016)
LysECD7-SMAP, LysECD7-flex-SMAP, LysECD7-rigid-SMAP	LysECD7	SMAP-29 (1-17, K2, 7, 13)	RKLRRLKRKIAHKVKKY	C-terminal	– GSAGSAAGSGEF AEAAAKEAAAKEAAAKA	*A. baumannii*	Antonova et al. (2020)
LysECD7-SMAP	LysECD7	SMAP-29 (1-17, K2, 7, 13)	RKLRRLKRKIAHKVKKY	C-terminal	–	*K. pneumoniae*, *A. baumannii*, *P. aeruginosa*, *Staphylococcus haemolyticus*, *Achromobacter* spp., *Burkholderia cepacia* complex and Haemophylus infuenzae	Vasina et al. (2024)
LysAB-vT2-fusion	LysAB-vT2	–	FILIVFVLIIAP	C-terminal	–	*A. baumannii*	Sitthisak et al. (2023)
Tha-PA90	PA90	Thanatin	GSKKPVPIIYCNRRTGKCQRM	N-terminal	–	*A. baumannii*	Lim et al. (2024)
LoGT-019 to LoGT-049	OBPgp279 and PVP-SE1gp146	PCNP	KRKKRKKRK	N-terminal	–	*A. baumannii*, *P. aeruginosa*, *E. coli*, *S. typhimurium*	Briers et al. (2014b)
Artilysin A Artilysin B	Endolysin A Endolysin B	PCNP	KRKKRKKRK	N-terminal	–	*H. pylori*	Xu et al. (2021)
5aa 10aa 15aa Mix	Lysep3	–	KRKRK KRKRKRKRKR KRKRKRKRKRKRKRK KRKRKFFVAIIP	C-terminal	–	*E. coli*	Ma et al. (2017)
Lysqdvp001-5aa, Lysqdvp001-10aa Lysqdvp001-15aa	Lysqdvp001	–	KRKRK KRKRKRKRKR KRKRKRKRKRKRKRK	C-terminal	–	*V. parahmolyticus*	Ning H. et al. (2021)
Lysep3-3 Lysep3-5 Lysep3-7 Lysep3-12a Lysep3-12b	Lysep3	–	ILP FFVAP FVFIFAP FILIVFVLIIAP FIVILIVFLIAP	C-terminal	–	*E. coli*	Yan et al. (2019)
Lys1S-L9P	LysSPN1S	KL-L9P	KLLKLLKKPLKLLK	C-terminal	GGGGS	*E. coli*, *P. aeruginosa*, *A. baumannii*	Son et al. (2023)
DS—PA90	PA90	DS4.3	RIMRILRILKLAR	N-terminal	–	*A. baumannii*	Lim et al. (2022b)
Lys-Li5 Lys-MSI Lys-Li5-MSI	LysECD7	Li5 MSI594 Li5-MSI	KNYSSSISSIRA GIGKFLKKAKKGIGAVLKVLTTG KNYSSSISSIRAGGGGSGGGGSGGGGSGIGKFLKKAKKGIGAVLKVLTTG	C-terminal	GGGGS	*A. baumannii*	Li et al. (2024)
OAM-rEnd2	rEnd2	Lipid oleylamine	(*Z*)-octadec-9-en-1-amine	–	–	*P. aeruginosa*	Oliveira et al. (2024)
Art-Bp7e6	Bp7e	–	PLTCASGVTTSYGIGFQAKY	N-terminal	–	*E. coli*	Sui et al. (2023)
ApoE23-LysKp84B (CHU-1) COG133-LysKp84B (CHU-2)	LysKp84B	ApoE23 COG133	LRKLRKRLVRLASHLRKLRKRLL LRVRLASHLRKLRKRLL	N-terminal	– GGGGS (GGGGS)_2_ (GGGGS)_3_ (GGGGS)_4_	*A. baumannii*, *E. faecalis*, *K. pneumoniae* *S. aureus*, *E. cloacae* and *P. aeruginosa*	Chen et al. (2024)
P361 P362 P371 P372	–	LeuA-P	RRLGRALRRVLRRLARLW	C-terminal	–	*E. coli*, *S. enterica*	Nie et al. (2021)

The design of AMP–endolysin fusion constructs plays a critical role in determining their ability to penetrate the outer membrane and reach the periplasmic space, where the endolysin exerts its catalytic activity on the peptidoglycan layer. Naturally occurring endolysins that exhibit antibacterial activity often contain a positively charged peptide domain at their C-terminus, suggesting that C-terminal fusion with antimicrobial peptides (AMPs) might be the preferred strategy. However, current studies explore peptide conjugation at both the N- and C-termini of lysins. N-terminal fusion of AMPs such as SMAP-29 (Briers et al., 2014a; Wang et al., 2021) or cecropin A (Abdelkader et al., 2022; Islam et al., 2022; Lim et al., 2022a; Yang et al., 2015) facilitates initial interaction with and disruption of the bacterial outer membrane, thereby enhancing periplasmic accessibility. Conversely, C-terminal fusions may sterically hinder membrane interaction or result in suboptimal orientation of the AMP moiety, potentially limiting transport efficiency (Antonova et al., 2020; Chen et al., 2021; Ma et al., 2017). To date, no consensus has been reached regarding which terminus should be modified to achieve optimal antimicrobial performance. It seems that the nature of the AMP and its physicochemical properties—including charge, hydrophobicity, amphipathicity, and length—critically influence the antimicrobial potency of the fusion construct. For instance, Artilysins containing the short, highly cationic AMPs have demonstrated broad-spectrum activity and membrane-permeabilizing efficiency, while Lysocins employing longer bacteriocins like Colicin–Lysep3 hybrids (Yan et al., 2017) show higher activity against specific strains but may require optimized linker design for maximal efficacy.

The inclusion of linker sequences between endolysins and fused peptides plays a critical role in modulating the bactericidal activity of engineered fusion constructs. In the study by Antonova et al., three SMAP-29 peptide-fused variants of the LysECD7 endolysin were compared: one without a linker, one with a flexible Gly-Ser-rich linker, and one with a rigid alanine-rich helical linker. The variant lacking a linker (LysECD7-SMAP) exhibited the highest antibacterial activity under physiological conditions (PBS and serum) and against stationary-phase *A. baumannii* cells. In contrast, the rigid linker construct (LysECD7-rigid-SMAP) showed significantly reduced activity, suggesting that linker rigidity can hinder optimal positioning or membrane interaction of the AMP domain. The flexible linker variant (LysECD7-flex-SMAP) retained intermediate activity, indicating that linker flexibility supports more favorable domain orientation and function (Antonova et al., 2020). The studies by Wang et al. and Chen et al. strongly support that short, flexible linkers preserve the structural integrity and enhance the synergistic activity between AMP and endolysin domains. Overly long or rigid linkers may lead to misfolding or spatial disconnection between functional domains, reducing efficacy (Chen et al., 2024; Wang et al., 2021). These results suggest that linker design-particularly its length, flexibility, and composition-directly influences the spatial arrangement and synergistic activity of fused domains. Thus, systematic linker optimization is essential for enhancing the functional performance of endolysin–peptide fusion constructs, especially when targeting gram-negative pathogens. Rational selection of AMP partners, linker length, and orientation should therefore be guided by the intended target spectrum, membrane composition of the pathogen, and delivery context.

#### 3.2.2 Modular fusion proteins

Modular fusion proteins are engineered biomolecules composed of distinct functional domains that are fused to create a single polypeptide with enhanced or novel properties. These proteins are designed by combining different modules, each responsible for a specific function, such as enzymatic activity, binding specificity, or signal transduction. The modular nature allows for precise customization, enabling fusion proteins to perform complex biological tasks efficiently. Fusion proteins can be designed for biomedical applications, including targeted drug delivery, immunotherapy, enzyme engineering, and synthetic biology. One common strategy in designing modular fusion proteins is the fusion of enzymatically active domains with cell-binding domains to improve specificity and efficacy, particularly in therapeutic applications (Lin and Liu, 2016; Yi et al., 2022).

The endolysin 1D10 was engineered as a fusion protein composed of four modules: catalytic domain—responsible for hydrolyzing the peptidoglycan in the bacterial cell wall, binding module—ensures specific binding to target bacteria, cationic peptide—cecropin A, which increases outer membrane permeability in gram-negative bacteria, and stabilizing domain—enhances thermal stability and resistance to proteolytic degradation. The modified lysin demonstrated increased thermal stability and proteolytic resistance, making it more effective under physiological conditions. Unlike traditional endolysins, which require membrane permeabilization, 1D10 exhibited direct bactericidal activity, degrading the bacterial cell wall even in the presence of an intact outer membrane. Studies confirmed that 1D10 activity depends on both the presence of cecropin A and the catalytic domain. Deletion analysis further defined the role of each module in the enzyme’s mechanism of action. The lysin effectively killed a range of MDR gram-negative bacteria, including *P. aeruginosa* and *K. pneumoniae*. Given its stability and unique killing mechanism, 1D10 represents a promising candidate for alternative antimicrobial therapies against antibiotic-resistant infections (Gerstmans et al., 2023).

In contrast, another study focused on the characterization and application of Plychap001, a single catalytic domain of the Lysqdvp001 endolysin, for controlling *V. parahaemolyticus* and its biofilms. Plychap001 effectively degraded *V. parahaemolyticus* cells, demonstrating strong bactericidal properties. The single endolysin domain reduced biofilm formation, highlighting its potential as an agent for biofilm-associated infections. The study emphasized Plychap001’s potential application in the food industry to combat Vibrio-related contamination (Wang et al., 2022). The addition of the D8 binding domain from the *Bacillus amyloliquefaciens* bacteriophage endolysin to *E. coli* bacteriophage endolysin Lysep3 significantly increased its ability to bind and degrade *E. coli* peptidoglycan. The modified Lysep3 exhibited stronger lytic activity against *E. coli* compared to the original enzyme (Wang et al., 2017). These studies suggest that fusion with specialized binding domains can enhance endolysin efficacy against gram-negative bacteria, offering a promising strategy for combating antibiotic-resistant infections.

#### 3.2.3 Innolysins: receptor-binding protein-endolysin fusions

Athina Zampara and her colleagues had conducted pioneering research on “Innolysins” engineered molecules designed to combat gram-negative bacteria by fusing bacteriophage endolysins with receptor-binding proteins (RBPs). This fusion enables endolysins to target specific bacteria, overcoming the outer membrane barrier that typically restricts access to the peptidoglycan layer. In their study, the researchers introduced the Innolysins concept by fusing the endolysin from bacteriophage T5 with its RBP, Pb5. Among the engineered constructs, Innolysin Ec6 demonstrated bactericidal activity against *E. coli*, reducing bacterial counts by approximately 1 log in bacterial counts. This antibacterial effect was dependent on the presence of the FhuA receptor, the specific target of Pb5 on *E. coli* cells. Furthermore, Innolysin Ec6 exhibited activity against other bacteria possessing FhuA homologs, such as *Shigella sonnei* and *P. aeruginosa*. Expanding the Innolysin approach, the researchers targeted *Campylobacter jejuni*, a leading cause of foodborne illnesses. The team identified an H-fiber from a CJIE1-like prophage in *C. jejuni* CAMSA2147, which functions as a novel RBP. By fusing this H-fiber to phage T5 endolysin, they created Innolysin Cj1, which exhibited antibacterial activity against various *C. jejuni* strains, achieving up to a 1.30 ± 0.21 log reduction in bacterial counts after 45 min of exposure at 20°C. Additionally, the application of Innolysin Cj1 on contaminated chicken skin at 5°C resulted in a 1.63 ± 0.46 log reduction of *C. jejuni* cells, demonstrating its potential for food safety applications (Zampara et al., 2021). The modular nature of Innolysins allows for customization by selecting different combinations of endolysins and RBPs, enabling the targeting of a broad spectrum of gram-negative pathogens. Zampara’s work represents a significant advancement in the development of novel antibacterial agents, offering a promising strategy to address the growing challenge of antibiotic-resistant gram-negative bacterial infections (Zampara et al., 2020, 2021).

#### 3.2.4 Lysocins: bacteriocin-endolysin fusions

Another innovative study introduces “Lysocins,” a novel class of bioengineered antimicrobial molecules that fuse bacteriophage lysins with bacteriocins—bacterial toxins inhibiting the growth of related bacterial strains. The study utilizes bacteriocins as a delivery mechanism to transport lysins across the outer membrane of gram-negative bacteria. The engineered Lysocin PyS2-GN4, which combines the bacteriophage lysin GN4 with the *P. aeruginosa* bacteriocin Pyocin S2 (PS2), exhibited high lytic activity against *P. aeruginosa*. *In vitro* studies showed that Lysocins effectively killed *P. aeruginosa*, disrupted biofilms, and had minimal endotoxin release, making them superior to conventional antibiotics in human serum. *In vivo* experiments in a mouse model confirmed that Lysocins could significantly reduce bacterial infections, suggesting their potential as an alternative therapeutic strategy (Heselpoth et al., 2019). Yan et al. (2017) investigated how the N-terminal and central domains of Colicin A, a bacteriocin secreted by *E. coli*, enhance the extracellular lytic activity of a Lysep3 endolysin. The fusion of Colicin A domains with Lysep3 enabled the enzyme to bypass the outer membrane of *E. coli*, facilitating direct bacterial lysis. The N-terminal and central domains of bacteriocin were crucial for membrane disruption, allowing the phage lysin to reach the peptidoglycan layer. The engineered Colicin-Lysep3 fusion exhibited enhanced bactericidal activity, showing promise for antibiotic-free treatment strategies against *E. coli*. Domains of the same bacteriocin were conjugated to *Campylobacter-*derived lysins. Although this lysin was not derived from a bacteriophage, the study confirmed that conjugating lytic enzymes with bacteriocin domains significantly improves activity (Liu P. et al., 2023). By leveraging the specific receptor-binding properties of bacteriocins, Lysocins selectively target and kill certain gram-negative pathogens. The combination of lysins’ peptidoglycan-degrading ability and bacteriocins’ membrane-penetrating properties significantly enhances antimicrobial efficacy, positioning Lysocins as a promising next-generation antibacterial approach.

## 4 Use of endolysins that can penetrate the cell wall of gram-negative bacteria to design membrane-active peptides

Until recently, only a few endolysins with intrinsic antibacterial activity against gram-negative species were known (Lim et al., 2014; Thandar et al., 2016; Larpin et al., 2018; Sykilinda et al., 2018; Raz et al., 2019; Bai et al., 2020; Kim et al., 2020; Chu et al., 2022; Euler et al., 2023). The antibacterial efficacy of endolysins against gram-negative bacteria depends on two key properties: (i) enzymatic activity, which enables degradation of the PG layer and (ii) membrane permeabilization, which facilitates endolysin translocation into bacterial cells. Several natural features enabling outer membrane permeabilization have been identified in endolysins: many endolysins contain highly positively charged C-terminal segments. Some endolysins possess one or more amphipathic helices, the C-terminal fragments of these endolysins enhance outer membrane permeability. Researchers hypothesize that amphipathic helices competitively displace divalent cations, which stabilize the LPS layer, leading to local outer membrane disruption and allowing endolysin to translocate into gram-negative bacteria. Once inside, the PG layer is degraded, resulting in bacterial lysis.

Several cationic peptides with bactericidal properties had been successfully isolated from whole endolysin proteins (Düring et al., 1999; Thandar et al., 2016; Li et al., 2021; Heselpoth et al., 2022; Szadkowska et al., 2022; Vázquez et al., 2022a, 2022b). Their activity was summarized in Table 3.

**Table 3:** Antimicrobial activity of peptides derived from phage endolysins of gram-negative bacteria.

Phage	Endolysin	Peptide sequence	Susceptible bacteria	Effective concentration	References
T4	T4	WDEAAVNLAKSRWYNQ	*E. coli*	0.1 μg/μl	Düring et al. (1999)
		PNRAKRVITTFRT		–	
RL-2015	PlyF307	NAKDYKGAAAEFPKWNKAGGRVLAGLVKRRK	*A. baumannii*	14.5 μM	Thandar et al. (2016)
ΦAB2	LysAB2	NPEKALEPLIAIQIAIKGMLNGWFTGVGFRRKR	*A. baumannii*	4–8 μM	Peng et al. (2017)
53	LysP53	MTMTTKRIFEHMRPNLTDSQVQALYKLMEKGAD	*A. baumannii*	100 μg/mL	Li et al. (2021)
JG004	Pae87	LNTFVRFIKINPAIHKALKSKNWAEFAKR	*P. aeruginosa*	20 μM	Vázquez et al. (2022a, 2022b)
–	PlyPa01	NAGDYAGAAEQFLRWNKAGGKVLPGLVRRRASERELFLGAA	*P. aeruginosa*	10 μg/mL	Heselpoth et al. (2022)
HB27	PhiK	RAPGWKSLAWLVRELRKHDSGLRLRLVRH	*A. baumannii* *E. coli*	3.9 μM 7.8 μM	Szadkowska et al. (2024)
PhiKo	LysC	KNLLRRIRRKLRNKFSRSDVIKTPKIVEVN	*A. baumannii*	7.8 μM	Szadkowska et al. (2022)

Düring et al. (1999) investigated the non-enzymatic microbicidal activity of lysozymes, particularly the T4 lysozyme. The study showed that amphipathic helix in the C-terminal region play a crucial role in antibacterial and fungistatic activities, even after heat denaturation. Synthetic peptides imitating to the C-terminal amphipathic domains exhibited microbicidal activity by disrupting bacterial and fungal cell membranes. However, they did not induce hemolysis in mammalian cells, suggesting selective antimicrobial action. The findings indicate that these peptides act by destabilizing microbial membranes while remaining non-hemolytic to mammalian (Düring et al., 1999). This work had inspired other researchers, who had focused on extracting short, active peptides from the endolysin sequences.

Thandar et al. (2016) engineered peptides derived from PlyF307, a phage lysin, targeting *A. baumannii*. The study identified a highly cationic C-terminal domain, which, after modification, exhibited enhanced antibacterial potency (Figure 3). The engineered peptides showed strong bactericidal activity *in vitro* and *in vivo,* particularly in murine skin infection models (Thandar et al., 2016). Researchers Li et al. (2021) characterized LysP53, a novel endolysin from an *A. baumannii* phage. Its first 33 amino acids (P104) were identified as a potent antimicrobial peptide capable of killing multidrug-resistant gram-negative pathogens. The antimicrobial peptide P104 exhibited bactericidal activity comparable to full-length LysP53, demonstrating high efficacy against *A. baumannii*, *P. aeruginosa*, *K. pneumoniae*, and *E. coli* (Li et al., 2021).

The paper Vázquez et al. (2022b) explores the structural and functional properties of the endolysin Pae87 from *P. aeruginosa* phage JG004, which were promising alternatives to conventional antibiotics, particularly against antibiotic-resistant *P. aeruginosa*. The crystal structure of Pae87 was determined, revealing the C-terminal region of Pae87 containing a positively charged AMP-like sequence (P87). This peptide can directly disrupt bacterial outer membranes, providing an additional mechanism for bacterial killing beyond enzymatic lysis. When its catalytic activity was knocked out (via mutations), Pae87 retained bactericidal activity. This suggests that the P87 region alone can act as an antimicrobial agent by permeabilizing bacterial membranes. The same authors improved the antibacterial activity of the peptide P87 through sequence modifications, resulting in the engineered peptide P88 (Vázquez et al., 2022a). The modifications increased its net charge and hydrophobicity, improving membrane interactions. P88 maintained activity against a wide range of *P. aeruginosa* clinical isolates, including multidrug-resistant strains, indicating its potential as a broad-spectrum antimicrobial agent and exhibited synergistic effects when combined with antibiotics like erythromycin, chloramphenicol, tetracycline, and azithromycin. P88 effectively reduced *P. aeruginosa* biofilms, although its activity against human A549 lung cells was higher compared to other antimicrobial peptides like melittin. The therapeutic index suggests potential for further optimization (Vázquez et al., 2022a).

PaP1 represents a highly potent, lysin-derived antimicrobial peptide with broad-spectrum activity, rapid bactericidal effects, and excellent biocompatibility. Derived from the C-terminal region of PlyPa01 endolysin, which naturally contains a cationic, amphipathic membrane-active region. Its topical use in treating chronic wound infections and biofilm-associated infections makes it a strong candidate for future clinical development. PaP1 permeabilizes the outer membrane of gram-negative bacteria and also disrupts the cytoplasmic membrane, leading to bacterial death. This dual mode of action eliminates the need for enzymatic peptidoglycan degradation, which is typical for full-length lysins. PaP1 was effective against a wide range of gram-negative and gram-positive pathogens, including: *P. aeruginosa*, *A. baumannii*, *E. coli*, *K. pneumoniae*, *S. aureus* (including methicillin-resistant *S. aureus*, MRSA), and *Enterococcus faecium* (including vancomycin-resistant *Enterococcus,* VRE). The peptide was effective in a murine model of burn wound infections, reducing bacterial loads of MRSA and MDR *P. aeruginosa,* and the enhancement in the efficacy of standard topical antibiotics (e.g., mupirocin, gentamicin), suggested synergistic potential. Combined with low toxicity to human cells (no hemolysis and low cytotoxicity toward neutrophils), PaP1 lysin constituted a safer alternative to existing membrane-targeting antimicrobials like polymyxins (Heselpoth et al., 2022).

Another study explores Intestinalin (P30), a cryptic antimicrobial peptide derived from the LysC enzyme of *Clostridium intestinale*. Interestingly, the amino acid sequence was similar to the endolysins of phages targeting *Thermus scotoductus,* isolated from Icelandic hot springs. This peptide was discovered within the N-terminal region of LysC, and its antimicrobial properties surpass those of the full-length enzyme. P30 represents a promising blueprint for the development of novel peptide-based antibiotics, particularly for difficult-to-treat infections and biofilm-associated diseases (Szadkowska et al., 2022). The same authors investigated PhiKo endolysin, an enzyme derived from the phiKo bacteriophage that infects the extremophilic bacterium *Thermus thermophilus* HB27 (Szadkowska et al., 2024). The research also identifies RAP-29, a cryptic lytic peptide hidden within the C-terminal region of PhiKo. The study provides a comprehensive molecular characterization of the PhiKo endolysin and the cryptic peptide RAP-29. While PhiKo endolysin is highly thermostable but has limited mesophilic activity, RAP-29 emerges as a potent, broad-spectrum AMP. RAP-29 demonstrates strong antimicrobial properties against gram-positive and gram-negative bacteria, making it a promising candidate for novel antimicrobial therapies. The study validates the concept of cryptic antimicrobial peptides hidden within lysins and proves that bioinformatics-driven peptide discovery can lead to novel antibiotic candidates. Such a promising perspective encourages further modifications to optimize stability, selectivity, and *in vivo* efficacy (Szadkowska et al., 2024).

The isolated peptides frequently demonstrated comparable or superior antibacterial activity to their full-length endolysins. Moreover, these peptides were effective *in vivo*, successfully treating bacterial infections and accelerating wound healing in murine models. These findings validate cryptic antimicrobial peptides as a novel antimicrobial strategy, emphasizing the therapeutic potential of lysin-derived peptides for next-generation antibacterial therapies. However, to date, the discovery of AMPs has mostly been based on their similarities to already-known microbicidal molecules. Introducing bioinformatics methods, including machine learning-based predictions, has accelerated potential AMP screening.

## 5 Using machine learning to design endolysins active against gram-negative bacteria

Computational methods and artificial intelligence are increasingly used to design and optimize endolysins for improved antibacterial activity. These approaches enable rapid modifications, functional predictions, and structural optimizations to enhance the efficacy of lysins against gram-negative bacteria. For example, VersaTile is an innovative modular synthetic biology platform designed to accelerate the engineering and optimization of lysins for antibacterial applications (Gerstmans et al., 2020). This platform allows for rapid assembly and screening of engineered lysins by combining different functional modules, such as cell wall-binding domains and enzymatic cleavage domains. The VersaTile platform created a diverse library of lysin variants, tested for antimicrobial activity against gram-positive and gram-negative bacteria. The study identified promising lysin candidates with enhanced stability, specificity, and bactericidal activity. For example, engineered variants targeting *S. aureus* and *K. pneumoniae* retained activity in serum and under high salt conditions, highlighting their therapeutic potential. VersaTile enables rapid hit-to-lead development, streamlining the discovery of highly effective engineered lysins for potential therapeutic applications (Gerstmans et al., 2020; Duyvejonck et al., 2021; Vander Elst et al., 2023).

The researchers (Tresnak and Hackel, 2023) conducted a comprehensive sequence–function analysis of lysins, evaluating their bactericidal efficiency, thermostability, and structural integrity. Using mutational scanning, they identified key amino acid residues responsible for optimal enzymatic activity and bacterial cell wall degradation. This study provided valuable insights into the structural determinants of lysin function, guiding the rational design of improved lysins with enhanced stability and potency. The findings contribute to the development of engineered lysins with broader antimicrobial activity and enhanced therapeutic potential (Tresnak and Hackel, 2023). The researchers (Zhang Y. et al., 2024) utilized machine learning and deep learning models to analyze large datasets of phage genomes and predict lysins with potential antibacterial activity. This artificial intelligence (AI)-driven approach enabled the discovery of previously unknown lysins, expanding the repertoire of phage-derived antimicrobial agents. Identified lysins were experimentally validated, demonstrating bactericidal activity against various bacterial pathogens, including antibiotic-resistant strains. The study highlights the power of AI in accelerating lysin discovery, offering a more efficient alternative to traditional experimental screening (Zhang Y. et al., 2024). The *in silico* design and development of novel chimeric lysins with high selectivity against *Salmonella* were tested. Using a computer-aided design approach, researchers engineered chimeric lysins by fusing different endolysins with antimicrobial peptides to enhance bacterial targeting and lytic activity. Various *Salmonella*-targeting endolysins were selected and modified through fusion with antimicrobial peptides to improve their binding affinity and lysis efficiency. Computational modeling predicted structural stability and binding interactions of the chimeras with *Salmonella* peptidoglycan. Molecular docking confirmed that the engineered lysins had a higher binding affinity to the *Salmonella* cell wall. The fusion peptides improved bacterial membrane interaction, suggesting potential for *in vitro* and *in vivo* applications. This study highlights the power of computational tools in designing novel chimeric lysins with enhanced antibacterial properties, paving the way for further experimental validation and therapeutic development (Nie et al., 2021).

The minireview by Bałdysz et al. (2024) provides a comprehensive overview of bioinformatic tools and databases relevant to lysin discovery, characterization, and engineering. The authors assessed the strengths and limitations of existing bioinformatics platforms used for predicting lysin structures, enzymatic functions, and bacterial targets. The study identified gaps in current computational tools, emphasizing the need for more specialized resources tailored to lysin research. Recommendations were provided on improving sequence annotation, structural modeling, and functional predictions to accelerate lysin-based antimicrobial development. The work stresses the importance of integrating computational and experimental approaches for better lysin optimization and therapeutic applications (Bałdysz et al., 2024).

An important consideration in machine learning-based lysin design is the selection of training datasets. Most current approaches rely exclusively on curated endolysin sequence databases, which offer domain-specific features relevant to muralytic activity. While this strategy ensures model focus and minimizes noise from unrelated protein classes, it also constrains the design space to variations within natural endolysin families. Expanding the training set to include non-endolysin proteins that also target peptidoglycan, such as bacteriocins, autolysins, or peptidoglycan-binding antimicrobial peptides, could enable the discovery of novel domain combinations and activity-enhancing motifs. These heterologous proteins may contribute unique structural elements or binding strategies that are not represented in phage-derived endolysins. However, inclusion of such diverse sequences would require careful curation, consistent functional annotation, and normalization of sequence features to prevent overfitting or functional ambiguity. A hybrid approach that incorporates well-characterized muralytic proteins from multiple biological sources may ultimately improve the robustness and generalizability of predictive models for engineered lysins.

The integration of AI-driven computational methods had revolutionized lysin engineering by enabling rapid modifications, functional predictions, and structural optimizations. Approaches such as machine learning, deep mutational scanning, and modular synthetic biology platforms like VersaTile have accelerated the discovery of engineered lysins with improved stability, specificity, and bactericidal activity. These advancements streamline antimicrobial development and open new therapeutic avenues for targeting antibiotic-resistant gram-negative bacteria.

## 6 Conclusions, challenges, and future directions

The main advantage of endolysins over conventional antibiotics is their high specificity against particular bacterial strains. Unlike broad-spectrum antibiotics, endolysins selectively target bacterial peptidoglycan, reducing collateral damage to the host microbiota or the induce in gene transduction. While research continues, studies on several endolysins, such as Tha-PA90 (Lim et al., 2024), ElyA1 and ElyA2 (Blasco et al., 2020), PA90 fused with DS4.3 (Lim et al., 2022b) have demonstrated no major toxicity or adverse effects *in vivo*, even at high concentrations. Recent preclinical and clinical evaluations further support the safety of endolysin-based therapeutics (ClinicalTrials.gov., 2017; Watson et al., 2019). The lysin CF-301 (exebacase) exhibited an excellent safety profile in animal models and early-phase clinical trials, with no cytotoxicity observed toward human cells or significant adverse events. Similarly, SAL200, a recombinant endolysin targeting *S. aureus*, was evaluated in a phase I clinical trial and found safe and well-tolerated in healthy volunteers (Jun et al., 2017). These findings highlight the strong potential of endolysins as safe antibacterial agents, although continued monitoring in advanced clinical phases is warranted. This feature makes them an attractive alternative to traditional antibiotics, particularly in an era of escalating antimicrobial resistance. Endolysins act significantly faster than traditional antibiotics, effectively lysing bacterial cells within minutes. Moreover, they function effectively in biofilms and mucosal environments, where conventional antibiotics tend to fail. However, the therapeutic application of endolysins against gram-negative bacteria remains a major challenge due to the presence of an impermeable outer membrane that limits access to the peptidoglycan layer. Various strategies had been developed to overcome this barrier, including fusion with AMPs, combination with outer membrane permeabilizers, and encapsulation within nanocarriers. While these approaches demonstrated promising results, further optimization is necessary to enhance their stability, reduce immunogenicity, and ensure targeted delivery *in vivo*.

One of the most significant breakthroughs in recent years had been the identification of membrane-active peptides hidden within endolysin sequences. These peptides exhibit independent antimicrobial activity, enabling them to permeabilize bacterial membranes and act synergistically with enzymatic lysis. The discovery of such cryptic AMPs had opened new avenues for the rational design of next-generation peptide-based therapeutics. Further research responds to the challenge of: (i) Optimizing peptide modifications to enhance stability, selectivity, and resistance to proteolytic degradation. (ii) Investigating structure–function relationships of cryptic AMPs to understand their interactions with bacterial membranes. (iii) Evaluating *in vivo* efficacy and safety profiles in clinically relevant animal models. (iv) Developing combination therapies where endolysin-derived peptides are paired with existing antibiotics or other antimicrobial agents to combat MDR pathogens.

Additionally, synthetic biology and AI-driven approaches are becoming increasingly valuable for optimizing endolysin-derived peptides. Machine learning models can predict peptide activity, stability, and toxicity, accelerating the design of novel antimicrobial agents. These AI-driven methods can also be applied to identify previously unrecognized antimicrobial sequences within lysin databases.

Endolysin-derived peptides represent a promising frontier in antimicrobial therapy, offering potent activity against MDR gram-negative bacteria. Future research should focus on refining their structure, improving delivery mechanisms, and ensuring safety and efficacy in clinical settings. With continued advancements in protein engineering and AI-assisted peptide design, these molecules have the potential to revolutionize the treatment of bacterial infections and provide a much-needed alternative to conventional antibiotics.
